# The Superior Advantage of Tapping at the Navel, Illustrated by a Case

**Published:** 1805-11-01

**Authors:** Ennalis Martin

**Affiliations:** Easton, (Maryland)


THE SUPERIOR ADVANTAGE OF TAPPING AT THE NAVEL, *illustrated by a Case, by* ENNALIS MARTIN, M. B. *of Easton, (Maryland) December,* 15, 1804.

PARACENTESIS, or tapping, is an operation generally considered the most simple and easy of all others in the art of surgery; and yet, I will venture to assert, there are few operators who have not felt some embarrassment in cases of ascites, as to the safety of performing this most imple operation. The physician and surgeon, in all such embarrassing cases, may console himself in this way: the case is a desperate one; the patient can, at all events, live but a short time, and perhaps the operation may hasten his end; as it is well known, its effects are not always to be guarded against by the most skilful; and though relief may be afforded, it can be only temporary. This is a very improper mode of reasoning, not to say cruel and inhuman. *Few and evil are the days of man.* To correct and lessen the evils of human life, is the duty of every individual; but that of a physician and surgeon is to lessen or relieve altogether the distresses of man arising from disease, and to preserve his life to the utmost span. Though the cause of disease cannot be removed, the pain and distress may be much alleviated, and life made comfortable a few weeks, months, or years, to the great consolation of friends and relatives. To the physician and surgeon it must afford no small satisfaction that he has done the utmost that could be done to relieve his patient; and that he has not stumbled upon the wrong method, when there was a right one untried. I consider the following case in point point to the above observations, and to the superior advantage of tapping at the navel.

Mrs. H. M. H—— was my patient twenty-one years ago. I knew her well, and have a pleasure in bearing testimony to her good sense, amiable qualities, and domestic virtues. When a child, she had suffered a small distortion in her spine, which grew with her growth, though it never could be said to have affected her general health. She married at the age of twenty-five years, and bore four healthy fine children, all of whom are now living. Some time previous to her marriage, she became affected with a pulmonic disease, which seemed to be so much fixed, as to excite a general belief among her friends, that she was consumptive. With much truth may it be asserted, the lancet was the sole means of preserving her life; of which she was so much convinced, that she always avowed herself a friend to the *depleting system*, though she had, for many years, outlived the use of it.

About this time two years, in the forty-fifth year of her life, the menses had stopped, and the abdomen began to swell, which induced her to think all was not right; but from the banters of her friends, and feeling no unusual indisposition, she was induced to think with them, that she might be pregnant, though the symptoms attendant on this occasion were not altogether such as she had formerly experienced. As she gradually increased in size from November to April, with gradually declining health, and several tumours beginning now to be perceptibly felt in the abdomen, she, as well as her friends, became concerned about her situation. Dr. Johnson was applied to, who expressed doubts, and desired that I might be called in consultation. We were both decidedly of an opinion her case was an ascites, accompanied with encysted tumours, and perhaps some of the viscera so much enlarged, particularly the omentum, as to make it difficult to distinguish the one from the other.

Under an impression that the disease proceeded from visceral obstructions, as well as a general vitiated habit, we began a mercurial course, aided with the most powerful diuretics, such as fol. digit. purp. but to our surprise we found the old fashioned sal. nit. more beneficial. Besides these capital remedies, others were used as symptoms seemed to require. As soon as the system was properly charged with mercury, absorption commenced, the abdomen subsided, and we had, in some measure, very pleasing prospects before us, as her general health was much

 mended:

mended: but those tumours, which were only felt by hard pressing on the walls of the abdomen before the water was absorbed, were now plainly seen as well as felt, and probably had rather increased than diminished in size. She now walked about the house, and occasionally rode out in a close carriage: but alas! as was expected, these pleasing prospects were of short duration. The abdomen began to swell. The same remedies were pushed to the utmost extent, but in vain. The fluctuation of water was more sensible to the touch than for months before; and the tumours were scarcely perceptible without hard pressing with the fingers. In short, the distention of the abdomen became very distressing and painful. No other alternative seemed now to remain than to attempt a perforation of the abdomen, and to draw off its fluid contents. She herself was anxious for tapping, or any thing which could afford her some respite from constant anxiety and pain. We were fully impressed that the operation ought to be performed; but the fear of wounding these *tumours*, which might not be hydatids, but an enlarged omentum, or some other *viscus*, put us to a stand. At length, finding nothing could be gained by delay, but that every thing was to be apprehended from it, we determined that the *tumours* should be shoved towards the navel, and that an incision should be made through the skin with a lancet, exactly half way, by *measurement*, between the navel and the spine of the os ilium, and that then the trocar should be cautiously introduced through the incision into the cavity of the abdomen. This plan was put into execution on the 29th day of October, 1808, without the least injury or accident, and ten pints only of a gelatinous fluid were evacuated, partly through the canula, and more through the wound between the canula and lips of the wound. After the evacuation of the fluid she was a little fainty, and complained of an uneasy painful sensation through the bowels; all which, in the space of an hour, went off, and she felt much relieved for several days, particularly from the painful distention of the abdomen, and a disposition to eject her aliment, which had been a constant attending symptom from the time the abdomen began to swell, and its contents to press upon the stomach.

In the course of a week, the abdomen began gradually to distend with water again, and, at the end of a fortnight, it was evidently more enlarged than before she was tapped, and was equally, if not more painful, which obliged us to think of a second operation. The wound was was not yet healed, and had rather the appearance of an ill-conditioned sore. Her situation would not admit of delay, and we had to fix on a spot as near to that first operated on as possible, as well as to avoid several blue veins thereabouts. On the 23d of November, the second operation was performed with the same caution, and twenty pints of water were drawn off, to her inexpressible relief, though, as before, she was sick and fainty. Now, as repeatedly before, diuretics, tonics, &c. &c. were prescribed, as well to keep up the appearance of doing something, as to prevent the accumulation of water, but to no purpose; the sufferings of our patient were daily increasing, and nothing less than a speedy evacuation of the contents of the abdomen held out a prospect of relief. Notwithstanding she possessed more than the usual fortitude of her sex upon all such trying occasions, a dread had seized her mind, and nothing but extreme pain could have induced her to submit to the operation. The first wound now looked worse than when the second operation was performed, and the second had a bad appearance, and we had to choose a spot between, and a little above the two first wounds. On the 7th of the following month (December), she was again tapped with great caution, but with the most alarming consequences. Syncope took place, and we had the dreadful mortification of thinking our patient was expiring under our hands. However, we were soon relieved from our consternation, by her revival, and though a small quantity of water was evacuated, she was much relieved, after experiencing considerable pain through the abdomen for two hours.

Every thing now proceeded as before for a fortnight; that is, in the course of a week, the abdomen gradually enlarged, with increasing pain from distention, and the usual ejection of food from the stomach. Nothing remained that afforded a chance for relief, but a discharge of the contents of the abdomen. We were loth to hazard the same result, or perhaps inevitable death. The navel had presented itself distended almost in diameter an inch. We reflected, that the only barrier between us and the contents of the abdomen was a little thin distended skin, cellular membrane hardly perceptible upon dissection, and the peritoneum stretched to nothing; and though this perforation through the linea alba, by the umbilical cord, should not be so large as it appeared to be by distention, there could not much injury arise from cutting a little upon the linea alba itself; at all events we should meet with no veins or arteries to plague us, and if we should cut this tendinous substance, it would probably heal as soon as the first wound by the trocar; but when our patient was told, that no other instrument was to be used than the lancet, and that this practice was sanctioned half a century ago, by an old and celebrated surgeon, Mr. Sharpe, she readily assented to it, notwithstanding we candidly acknowledged we had never performed the operation in that way, nor seen it performed by others. It must be confessed, we had some scruples ourselves on this occasion, because we could not find, by the most diligent research, that the more modern improvements in surgery had authorized this mode of tapping. However, the dread of our patient, as well as our own apprehensions of the consequences, judging from the effects of the last operation, determined us to deviate from the beaten track of half way *measurement*. With some degree of confidence, she was laid partly on her side, and with a common lancet, a puncture was made through the navel, with as much, if not more ease, than a vein could have been opened in the arm. The water flowed in a stream, fully as large as a goose quill, while our patient expressed her surprise and satisfaction, declaring she was scarcely sensible that any thing had been done. "Bless me," said she, "why did you not try this method before? I shall never be under such dread again! I feel easier already!" It appeared as if less pressure was necessary to evacuate the waters; and what seemed very unaccountable, she experienced hardly any of those uneasy painful sensations which had been consequent on all the previous operations. As soon as the water ceased to flow, the lips of the wound closed together, and healed by the first intention in two days, by no other application than that of dry lint, and a plaster of basilicon.

Though now her health began to improve, and all the symptoms to wear a more favourable aspect, she was regularly tapped in this way once every fortnight, with the certain prospect of relief, losing each time from sixteen to twenty-two pints of water, until some time in April, 1804, when we began to date a gradual declension in her general health; a decrease in the quantity of water drawn off each time, and a perceptible increase in the size of the hydatids, from whence there was a more constant compression on the stomach, and, of course, less aliment lay there to answer the purposes of nutrition. Her flesh was very sensibly wasting away, and though we continued to repeat repeat the operation every fortnight, less benefit was obtained, and less water evacuated each time. What was very distressing to herself, as well as painful to us to observe, after drawing off all that could be pressed out, her size was scarcely diminished. Finding that the benefits arising from tapping would soon be at an end, unless the contents of the hydatids could be emptied, we now began to turn our attention to that desirable object, somewhat a ware of danger, and apprehending difficulties from their probable consistence. Reflecting that time was pressing hard upon us, and that none was to be lost, we determined, with the approbation of our patient and her friends, to tap her as usual at the navel, and while the waters were flowing, to examine with a probe one which lay directly over that part; and then, if adviseable, to perforate it with the trocar. Every thing appearing plain, and without hazard, I introduced the trocar through the puncture, and pushed it into the hydatid, then withdrew the perforator, which was followed slowly by a white gelatinous substance, which seemed to hang from the canula, and, in a little time, to cease to flow at all, which induced us to take hold of it with our fingers, and to pull at it. The canula was withdrawn, and again introduced, but all to no purpose. The air had coagulated this tough viscid matter, and made it a perfect gluten, which almost entirely closed up the orifice. The hydatid had come in contact with the internal lips of the wound, and produced a constant oozing of a thin fluid for many weeks, and thus effectually prevented it from healing by the first intention, which it uniformly had done before in forty-eight hours after each operation. Our distress may be readily conceived on this trying occasion, but that of our patient was mortifying in the extreme. She complained most piteously, that we had stopped the only tolerable avenue to a temporary relief! For five or six months she had enjoyed some comfortable days, but now, alas! she was suddenly deprived of all hope of even a temporary nature! Let it not be supposed this amiable lady was so much attached to life as to indulge an unreasonable hope. She had that natural desire, which inhabits the breast of every human being, of living free from pain. She had also endearing connections, and would willingly have spun out the tender thread of life a little longer; but at the same time, she had too much good ense and reflection not to know that she had, for a long time, been hastening to that " bourn from whence no

 traveller traveller returns." Patience and resignation had to her become habitual virtues; but when pain and affliction were constantly accumulating, it was natural to desire and to seek for relief.

Though we had now done what we thought would, probably, have been most conducive to the prolongation of the life of our patient, we were convinced we had taken a wrong step to effectuate that desirable object, and what was also very mortifying, we extracted only a few pints of water, not half the quantity which might have been drawn off, if we had not been so anxious to do more than heretofore. We shall certainly stand excused for going thus far, when it is considered that all the animal functions had almost entirely lost their energy, and scarcely a spark of the vital principle was remaining, that was not oppressed by what we wished to remove. If we could have succeeded in our grand design, we certainly should have arrested the strong arm of death a few months longer, though it must be obvious, our efforts could not have prevailed against the power of disease.

From the last date to the sixth of July, we remained little more than idle spectators of a scene, which we could only lament, and administered palliatives, which were constantly rejected by that organ, from which the animal system receives all its supplies of nourishment and strength. She now begged us to try any method to give some temporary respite from pain, which had become more aggravated. Accordingly, on the sixth day of July, we ventured the sixteenth time on the spot, where we had set out from on the 29th of October, 1803. But, alas! the waters were too gelatinous to flow, or were enveloped in their own membranes, forming hydatids, which we were, by former experience, warned from meddling with. At most we did not obtain more than five or six pints of a gelatinous fluid. Our labours were now at an end, and the *last enemy* our afflicted patient had to contend with *was death,* which cut the tender thread of life on the second day of August, 1804.

